# Editorial: Women in molecular neuroscience

**DOI:** 10.3389/fnmol.2024.1422589

**Published:** 2024-05-14

**Authors:** Eva Žerovnik

**Affiliations:** ^1^Department of Biochemistry and Molecular and Structural Biology, Jožef Stefan Institute, Ljubljana, Slovenia; ^2^Jožef Stefan's International Postgraduate School, Ljubljana, Slovenia

**Keywords:** multiple sclerosis, autoimmune disorder, pituitary adenylate cyclase-activating peptide (PACAP), brain-derived neurotrophic factor (BDNF), PLC-gamma-Ca+2 pathway, cystatin B, oxidative stress

Women in Science have been for a long time perceived as equal to men; no one objects they are as successful as their men colleagues in studies and research. In the real world, however, there might still be some barriers that women encounter, especially to achieve leadership positions. To support their developments, this Research Topic welcomed female contributions to molecular neuroscience, either, the lead and/or corresponding authors. Stress was put on early career researchers, teaming up with senior female colleagues. Different country regions were represented. Some papers give general perspectives on a specific field of research inspired, started, or sparked by a woman researcher, other papers describe molecular neuroscience studies led by women. The authors are from different countries and continent, all well-established women scientists.

The paper by Barateiro et al., from the Faculty of Pharmacy, University of Lisbon, Portugal, entitled “*Women in the field of multiple sclerosis: how they contributed to paradigm shifts*” (ed. by E.Ž), gives perspectives on the paradigm shift in the multiple sclerosis (MS) field of research as performed by women. Authors claim: “History is full of women who made enormous contributions to science. While there is little to no imbalance at the early career stage, a decreasing proportion of women is found as seniority increases.” The authors emphasize the new insights on MS sparked by remarkable women that have shifted paradigms by pointing to cognitive impairment, white and gray matter pathology, immune response, and the gut-brain interplay on MS diagnosis, progression, and/or therapy.

**MS is an autoimmune disorder of the central nervous system** (CNS), with evident paradigm shifts in the understating of its pathophysiology. In MS the immune system becomes overactivated and attacks the myelin sheath surrounding axons. The resulting demyelination disrupts the communication signals to and from the CNS. Classically, MS was viewed to cause mostly physical and motor disabilities. However, it is now recognized that cognitive impairment affects more than 50% of MS patients. Another shifting paradigm was the involvement of **gray matter in MS** pathology, formerly considered to be a white matter disease. In accordance with **the gut-brain connection** of brain pathologies, the gut microbiota were shown to influence not only different susceptibilities to MS pathology, but it can also modulate the course of disease. Not at least, the role of microRNAs has been investigated in MS, either as potential biomarkers or therapeutic agents.

In the paper by Cao et al. from the Vietnam National University, Ho Chi Minh City, Vietnam “*Plasma cell-free RNA profiling of Vietnamese Alzheimer's patients reveals a linkage with chronic inflammation and apoptosis: a pilot study*” (ed. by Ö.S.) profiles of cfRNA in Caucasian AD patients have been investigated. Their study examined the gap and contributed to the development of point-of-care AD diagnosis. Namely, **circulating cell-free RNA (cfRNA) is a potential hallmark for early diagnosis of Alzheimer's Disease (AD)** as it construes the genetic expression level, giving insights into the pathological progress from the outset.

The paper by Wang et al. from Chengdu University of Traditional Chinese Medicine, Chengdu, China entitled “*Brain structural and functional changes during menstrual migraine: relationships with pain*”, (ed. by Ö.S), **menstrual migraine (MM)** was explored. The authors investigated gray matter volume and functional connectivity alterations in patients with MM. Their results suggest that the anterior cingulate cortex (ACC) may be an important biomarker in MM, and its structural and functional impairments are significantly associated with the severity of pain and pain-related impairment of emotion in patients with MM.

“*The role of primed and non-primed MSC-derived conditioned media in neuroregeneration*” was investigated by Hudakova et al. from the University of Veterinary Medicine and Pharmacy in Kosice, Košice, Slovakia and Institute of Neuroimmunology, Slovak Academy of Sciences, Bratislava, Slovakia (as edited by Jolanta Dorszewska). With growing **significance in nervous system repair**, mesenchymal stem cell-derived conditioned media (MSCCM) have been used in cell-free therapies in regenerative medicine. However, the immunomodulatory and **neuroregenerative effects of MSCCM** and the influence of priming on these effects are still poorly understood. The results highlight the potential of primed and non-primed MSCCM as a useful therapeutic tool for neurodegenerative diseases.

In the review paper written by one of the Guest Co-editors, Žerovnik from Jožef Stefan Institute, Ljubljana, Slovenia “***Human stefin B****: from its structure, folding, and aggregation to its function in health and disease*” (as edited by Andrei Surguchov) structural, functional and pathological sides of human stefin B (cystatin B) were described. Mutations in the gene for **cystatin B cause progressive myoclonic epilepsy type 1 (EPM1)**, which also is, apart from epileptic syndrome, a neurodegenerative disorder. Function-wise, human stefin B primarily acts as a cysteine cathepsin inhibitor, however, it also exhibits alternative functions. It has been shown that stefin B is oligomeric in cells and that it has a specific role in the physiology of the synapse and in vesicular transport. It plays a **protective role against oxidative stress**, likely via reducing mitochondrial damage and reactive oxygen species (ROS). Lack of stefin B results in NLRP3 inflammasome activation, followed by increased inflammation and ROS. Primarily the cytosolic protein, it exerts an important role in the nucleus, where it prevents cleavage of the N terminal part of histone 3 by inhibiting cathepsins L and B, regulating transcription and cell cycle.

The paper by Slabe et al. from the Faculty of Medicine, University of Ljubljana, Slovenia and the Institute of the Royal Netherlands Academy of Arts and Sciences, Amsterdam, Netherlands, respectively, “*Increased pituitary adenylate cyclase-activating peptide genes expression in the prefrontal cortex in schizophrenia in relation to suicide*” (ed. by E.Ž), discusses a role of **pituitary adenylate cyclase-activating peptide (PACAP**) in suicide victims. PACAP is a stress-related neuropeptide that is produced in several brain areas. It acts by 3 receptors: PACAP type-1 (PAC1), vasoactive intestinal peptide (VIP) −1 and −2 (VPAC1 and VPAC2). Data on polymorphisms in PACAP and PAC1 indicate a relationship of the **PACAP system with schizophrenia (SCZ)**. In their study, the dorsolateral prefrontal cortex (DLPFC) and anterior cingulate cortex (ACC) of 35 SCZ patients and 34 matched controls were included. Changes in expression in PACAP-related genes in relation to **SCZ, suicide and gender** were observed. An increase in all PACAP-related genes was present in SCZ-N male patients compared to SCZ-N females^1^. There was a higher PACAP-related gene expression observed in SCZ patients in the ACC and in suicide victims with SCZ in the DLPFC.

Furthermore, original research was performed by the group of Francisca C. Bronfman from the Faculty of Medicine and Faculty of Life Sciences, Universidad Andres Bello, Santiago, Chile (ed. by E.Ž) “***PLC-gamma-Ca*+*2*** ***pathway*** *regulates axonal TrkB endocytosis and is required for long-distance propagation of BDNF signaling*” (Moya-Alvarado et al.). In this work, **brain-derived neurotrophic factor (BDNF)** and its **tropomyosin receptor kinase B (TrkB)** were studied. These proteins regulate dendritic growth and maintenance in the central nervous system (CNS), Namely, after binding of BDNF, TrkB is endocytosed into endosomes and continues signaling within the cell soma, dendrites, and axons. The authors have shown that the activity of PLC-γ is required for BDNF-dependent TrkB endocytosis, suggesting a role for the TrkB/PLC-γ signaling pathway in axonal signaling endosome formation.

The group of Irena Smaga from the Maj Institute of Pharmacology Polish Academy of Sciences, Kraków, Poland contributed original research about post-delivery **depression in the offspring**: “*A maternal high-fat diet during pregnancy and lactation induced depression-like behavior in offspring and myelin-related changes in the rat prefrontal cortex*” (Frankowska et al.) (ed. by Ö.S.).

All in all, we with the Co-Editor are proud for the women contributors, who took the challenge to present their important works.

## Author contributions

EŽ: Conceptualization, Writing – original draft, Writing – review & editing.

## Footnotes

^1^SCZ-normal means patients with schizophrenia who died normally.

